# Transplantation of Bone Marrow-Derived Mononuclear Cells Improves Mechanical Hyperalgesia, Cold Allodynia and Nerve Function in Diabetic Neuropathy

**DOI:** 10.1371/journal.pone.0027458

**Published:** 2011-11-18

**Authors:** Keiko Naruse, Jun Sato, Megumi Funakubo, Masaki Hata, Nobuhisa Nakamura, Yasuko Kobayashi, Hideki Kamiya, Taiga Shibata, Masaki Kondo, Tatsuhito Himeno, Tatsuaki Matsubara, Yutaka Oiso, Jiro Nakamura

**Affiliations:** 1 Department of Internal Medicine, School of Dentistry, Aichi-Gakuin University, Nagoya, Japan; 2 Futuristic Environmental Simulation Center, Research Institute of Environmental Medicine, Nagoya University, Nagoya, Japan; 3 Department of Removable Prosthodontics, School of Dentistry, Aichi-Gakuin University, Nagoya, Japan; 4 Diabetes Center, Aichi Medical University, Nagakute, Aichi, Japan; 5 Department of Endocrinology and Diabetes, Nagoya University Graduate School of Medicine, Nagoya, Japan; The University of Hong Kong, Hong Kong

## Abstract

Relief from painful diabetic neuropathy is an important clinical issue. We have previously shown that the transplantation of cultured endothelial progenitor cells or mesenchymal stem cells ameliorated diabetic neuropathy in rats. In this study, we investigated whether transplantation of freshly isolated bone marrow-derived mononuclear cells (BM-MNCs) alleviates neuropathic pain in the early stage of streptozotocin-induced diabetic rats. Two weeks after STZ injection, BM-MNCs or vehicle saline were injected into the unilateral hind limb muscles. Mechanical hyperalgesia and cold allodynia in SD rats were measured as the number of foot withdrawals to von Frey hair stimulation and acetone application, respectively. Two weeks after the BM-MNC transplantation, sciatic motor nerve conduction velocity (MNCV), sensory nerve conduction velocity (SNCV), sciatic nerve blood flow (SNBF), mRNA expressions and histology were assessed. The BM-MNC transplantation significantly ameliorated mechanical hyperalgesia and cold allodynia in the BM-MNC-injected side. Furthermore, the slowed MNCV/SNCV and decreased SNBF in diabetic rats were improved in the BM-MNC-injected side. BM-MNC transplantation improved the decreased mRNA expression of NT-3 and number of microvessels in the hind limb muscles. There was no distinct effect of BM-MNC transplantation on the intraepidermal nerve fiber density. These results suggest that autologous transplantation of BM-MNCs could be a novel strategy for the treatment of painful diabetic neuropathy.

## Introduction

Diabetic neuropathy is the most common complication of diabetes. The abnormal peripheral sensations (skin or deep tissues) show great variety, such as paresthesia, allodynia, hyperalgesia, and spontaneous pain. Especially, chronic neuropathic pain is present in 3 to over 20% of diabetic patients [1]. Pharmacological treatments such as anti-depressants and anti-convulsives are effective but the effects are partial in many cases, and thus many patients have a marked reduction in the quality of life [2]. The relief from symptoms of diabetic neuropathy is, therefore, still an important issue for many clinicians.

It has been reported that diabetic animal models show pain-related behaviors that mimic symptoms of painful diabetic neuropathy in humans [3]. Hyperalgesic behaviors are observed in the early stage of diabetes of several animal lines such as streptozotocin (STZ) diabetic rats and mice, BB/Wor rats, Zucker diabetic fatty rats, and ob/ob mice [4]–[6]. Using these animal models, many studies have been done to understand of the mechanisms of painful diabetic neuropathy. Treatment with antioxidants, such as α-lipoic acid [7], taurine [6] and a poly(ADP-ribose) polymerase (PARP) inhibitor [8], or aldose reductase inhibitors [9], [10] ameliorated mechanical and thermal hyperalgesia in diabetic rats. Treatment with C-peptide also improved diabetic mechanical and thermal hyperalgesia, and the improvement was accompanied by recovery from the morphometric abnormalities and decreased contents of several neurotropic proteins in the peripheral nerves [4]. α-Lipoic acid and an aldose reductase inhibitor are available for the treatment of diabetic neuropathy in some countries and some other drugs are in clinical trials [11], but more powerful therapies are needed for the treatment of painful diabetic neuropathy not only for the relief from pain but also to improve nerve functions.

The disturbance of peripheral blood flow is one of the major pathological causes of diabetic neuropathy [12]. Impairments of cutaneous endothelium-related vasodilatation, C-fiber-mediated vasoconstriction, and epineural blood flow in the sural nerve were observed in diabetic painful neuropathy [13], [14]. Several clinical studies have shown that transplantation of bone marrow-derived mononuclear cells (BM-MNCs) into the skeletal muscles can be an effective treatment for ischemic limbs, because it enhances post-ischemic neovascularization and increases vascular blood flow [15], [16]. The bone marrow is known to contain larger numbers of progenitor or stem cells such as endothelial progenitor cells and mesenchymal stem cells than peripheral blood [17], [18]. BM-MNCs secrete potent angiogenic ligands (basic FGF, VEGF, angiopoietin-1) and cytokines (IL-1β and TNF-α), and are also incorporated into the local neovascularization. A benefit of the transplantation of freshly isolated MNCs is that autologous MNCs can be isolated in a closed cavity, not only in *ex vivo* culture conditions. We have previously demonstrated that the transplantation of cultured endothelial progenitor cells or mesenchymal stem cells improved diabetic neuropathy in STZ rats [19], [20]. Transplantation of bone marrow or peripheral blood MNC also improved the delay of nerve conduction velocity and nerve blood flow in STZ rats [21], [22]. Taken together, MNCs could be a promising cell source for the treatment of diabetic neuropathy, including diabetic hyperalgesia, because of their secretion of angiogenic ligands and cytokines involved in vasculogenesis. In the present study, therefore, we examined whether transplantation of freshly isolated BM-MNCs improved mechanical hyperalgesia and cold allodynia in STZ diabetic rats. We found, for the first time, that intramuscular transplantation of BM-MNCs ameliorated the diabetic neuropathic pain, accompanied with the functional recovery of the peripheral nerves and increased tissue blood flow.

## Methods

### Animals

Male Sprague-Dawley (SD) rats were obtained from Japan SLC, Inc. (Shizuoka, Japan) at 6 weeks of age. All rats were housed in individual cages under controlled temperature (24±1.0°C) and on a 12 h light/dark cycle, and were given standard laboratory rat chow with water ad libitum. Diabetes was induced by a single intraperitoneal injection of freshly dissolved STZ (Sigma Chemical Co., MO, USA) (60 mg/kg body weight in 0.9% sterile saline) to rats after an overnight fast. Diabetes was identified by polydipsia, polyuria and by measuring the non-fasting serum glucose concentration 1 week after the injection of STZ. Rats with a blood glucose level above 12.5 mM were considered to be diabetic and were used in the experiments. Age-matched male SD rats were used as control animals. All experimental protocols were conducted according to the Regulations for Animal Experiments in Nagoya University, and were approved by the Institutional Animal Care and Use Committees of Nagoya University and Aichi Gakuin University.

### Bone marrow-derived mononuclear cells

Bone marrow was taken from the femoral and tibial bones in 6-week-old male SD rats and was suspended in phosphate buffered saline (PBS). BM-MNCs were isolated using the Histopaque-density centrifugation method. The MNC layer was collected, washed twice with PBS, and suspended in 0.9% saline with 0.5% bovine serum albumin (BSA).

### Transplantation of BM-MNCs

Two weeks after the STZ injection, the diabetic rats were deeply anesthetized with pentobarbital (50 mg/kg body weight, intraperitoneally) and were transplanted with BM-MNCs into the hind limb skeletal muscles. The MNC suspension (0.5 ml in total, 1×10^6^ cells) was injected into 10 points in the unilateral femoral quadriceps, femoral biceps and soleus muscles using a 26-gauge needle. Saline (0.5 ml in total) was also injected into the contralateral hindlimb skeletal muscles as the control. In some behavioral tests, saline (0.5 ml in total) was injected into the unilateral hind limb skeletal muscles in another group of diabetic control rats.

### Measurements of nociceptive behaviors

All behavioral tests were performed in a blinded fashion. Baseline behavioral tests were carried out to assess the degree of mechanical hyperalgesia and cold allodynia before the STZ injection, and the same test was repeated at 1–2 weekly intervals for 4 (mechanical hyperalgesia) to 6 (cold allodynia) weeks, beginning 2 weeks after STZ injection. Each rat was placed individually beneath an inverted transparent plastic cage (11×17×11 cm) with a wire mesh bottom and habituated to the test chamber for at least 30 min before the measurements.

#### 1. Mechanical hyperalgesia

Pain-related behavior induced by mechanical stimulation was measured with two types of homemade von Frey hairs (VFHs, diameter: 0.5 mm, bending force 93 and 197 mN). Each VFH was applied perpendicularly ten times (once every 2–3 sec) to the unilateral mid-plantar hind paw, and the number of foot withdrawals was then counted. Stimulation of normal human skin with these VFHs elicits a sensation of painful pricking. A significant increase in the frequency of foot withdrawals in response to this mechanical stimulation was interpreted as mechanical hyperalgesia.

#### 2. Cold allodynia

Behavioral responses to innocuous cold stimulation were tested by the acetone drop test [23]. A 50 µl droplet of acetone was applied to the unilateral mid-plantar hind paw using a syringe connected to a thin polyethylene tube. Acetone application was repeated ten times at intervals of approximately 3 min between each application, and the number of foot withdrawals was then counted. A brisk foot withdrawal response after the spread of acetone over the plantar surface of the paw was considered a sign of cold allodynia.

### Sciatic nerve conduction velocities

Two weeks after BM-MNC transplantation, sciatic nerve conduction velocities were measured. Rats were deeply anesthetized with pentobarbital and placed on a heating pad in a room maintained at 25°C to ensure a constant rectal temperature of 37°C. Motor nerve conduction velocity (MNCV) between the ankle and sciatic notch, and sensory nerve conduction velocity (SNCV) in the sciatic nerve between the ankle and knee were measured using a non-invasive procedure. MNCV and SNCV were determined with a Neuropak NEM-3102 instrument (Nihon-Koden, Osaka, Japan) by methods described previously [24].

### Sciatic nerve blood flow (SNBF)

Two weeks after BM-MNC transplantation, SNBF was measured by the hydrogen clearance technique with an analog recorder BW-4 (Biochemical Science, Kanazawa, Japan) and electrolysis tissue blood flow meter RBA-2 (Biochemical Science, Kanazawa, Japan), as described previously [25].

### Capillary density in skeletal muscles

Two weeks after BM-MNC transplantation, rats were killed with an overdose of pentobarbital. The soleus muscles of both hind limbs were removed and fixed in a 4% paraformaldehyde solution overnight. The fixed materials were embedded in paraffin and cut into 5-µm sections for immunohistochemical staining with primary antibody, as previously described [19]. The sections were incubated overnight at 4°C with the primary antibody (anti-vWF polyclonal antibody, DAKO Japan, Tokyo, Japan) diluted 1∶600 and subsequently stained using the Simplestain rat system (Nichirei, Tokyo, Japan) according to the manufacturer's instructions. The negative control was performed by omitting anti-factor VIII antibody. The capillary endothelial cells were counted under light microscopy (×200) to determine the capillary density. To avoid overestimating the capillary density due to muscle atrophy or underestimating it due to interstitial edema, the capillary density was expressed as the capillary/muscle fiber ratio.

### mRNA expressions of skeletal muscles

Total RNA was extracted from the frozen samples of soleus muscles using using TRIzol Reagent (Invitrogen, Carlsbad, CA), according to the manufacturer's instructions. Starting from 500 ng RNA, cDNA was synthesized using ReverTra Ace (Toyobo, Osaka, Japan), according to the manufacturer's instructions. Primers and probes for basic FGF, NT-3, and 18S rRNA for the endogenous control were purchased from Taqman Gene Expression Assays (Applied Biosystems, Foster City, CA). Real-time quantitative PCR was performed and monitored using ABI Prism 7000 (Applied Biosystems). Relative quantity was calculated by the 2[DELTA]^[DELTA]ct^ method [26].

### Intraepidermal nerve fiber density (IENFD)

IENFD was assessed as described previously with minor modification [27]. Briefly, at the end of the experiments, rats were sacrificed with an overdose of pentobarbital. Both footpads were removed and fixed in a 4% paraformaldehyde solution overnight. The fixed materials were embedded in paraffin and cut into 5-µm sections. The sections were incubated overnight at 4°C with the primary antibody (anti-PGP9.5 antibody, Abnova, Taipei City, Taiwan) diluted 1∶400 and subsequently stained using the Simplestain rat system (Nichirei) according to the manufacturer's instructions. The negative control was performed by omitting anti-PGP9.5 antibody. Intraepidermal nerve fiber profiles were counted blindly by 3 independent investigators under light microscopy (×200) and the average values were used.

### Statistical analysis

All group values were expressed as means ± standard error of the mean (SEM). Two-way analysis of variance (ANOVA) with repeated measures or one-way ANOVA, and Dunnett's post-hoc test were utilized to analyze the influence of STZ injection and BM-MNC transplantation on pain behaviors, as appropriate. Statistical analyses for other tests were made by one-way ANOVA followed by the Bonferroni correction for multiple comparisons. Differences were considered significant at the *P*<0.05 level.

## Results

### Changes in body weights gain and blood glucose concentrations

Diabetic rats showed significant decreases in body weight gain and significant increases in blood glucose concentrations compared with normal rats (Table S1). The transplantation of BM-MNCs into the unilateral hind limb skeletal muscles did not change the body weights and blood glucose concentrations in the diabetic rats.

### Effects of BM-MNCs on mechanical hyperalgesia

**Figure 1:**
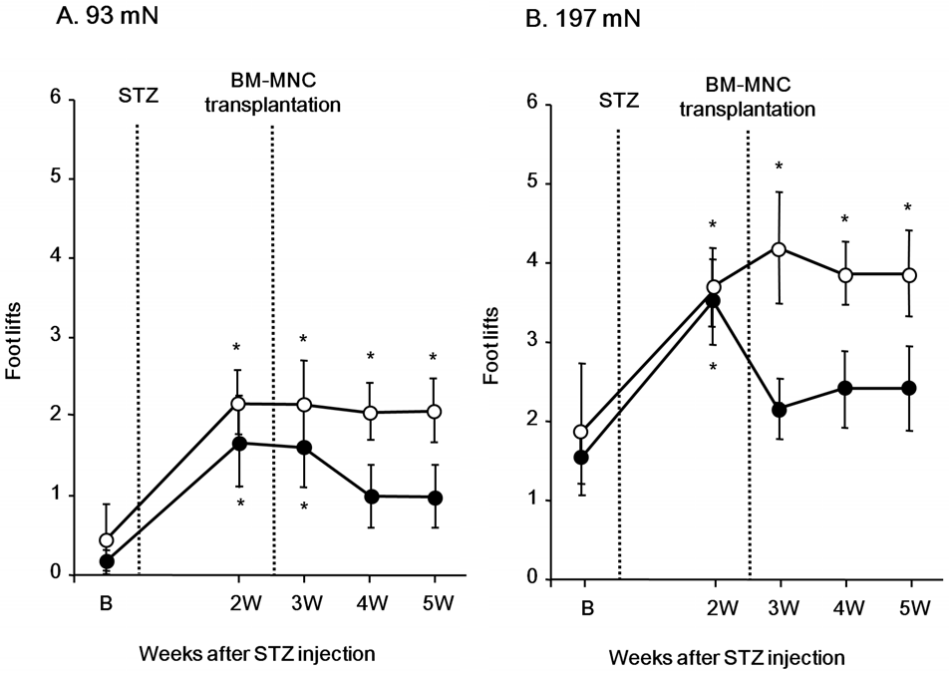
Inhibitory effects of BM-MNC transplantation on mechanical hyperalgesia induced by STZ administration. Number of foot withdrawals in response to the 93 (A) and 197 mN (B) von Frey hair (VFH) stimulation in diabetic rats is shown. Data are presented as mean ± SEM. Vertical axis, number of foot lifts; horizontal axis, order of the test (weeks after STZ injection). Broken line shows the time point of STZ administration and BM-MNC transplantation. In the treatment group (solid circles), BM-MNC injection was carried out just after the measurements at 2 weeks (2W) after the STZ injection (n = 8). In the control group (open circles), saline injection was done (n = 6). Repeated measures two-way ANOVA identified a significant difference in the day-effect between BM-MNC transplanted and control group. *P<0.05 compared with before (B) STZ injection (one-way ANOVA with Dunnett's tests).

Figure 1 shows the effects of BM-MNC transplantation on mechanical hyperalgesia in STZ-induced diabetic rats. Intraperitoneal administration of STZ elicited foot withdrawal with significantly increased frequency in the diabetic control rats. Namely, the number of foot withdrawals in response to the VFH stimulation (93 and 197 mN) was significantly increased 2 weeks after STZ injection, and the level remained high for another 3 weeks (93 mN: F_(4,20)_ = 3.73; 197 mN: F_(4,20)_ = 3.82, for both *P*<0.05). This implies that STZ-induced diabetes shows long-lasting mechanical hyperalgesia. In the BM-MNC transplanted group, a significant increase in the withdrawal response 2 weeks after STZ injection was also observed. Interestingly, the STZ-induced increase in the withdrawal response was inhibited after BM-MNC transplantation compared with that in the control group (93 mN: group, F_(1,12)_ = 3.11, *P*>0.05; day, F_(4,48)_ = 6.96, *P*<0.005; interaction, F_(4,48)_ = 0.48, *P*>0.05; 197 mN: group, F_(1,12)_ = 5.89, *P*<0.05; day, F_(4,48)_ = 5.76, *P*<0.001; interaction, F_(4,48)_ = 1.79, *P*>0.05), and the number of foot withdrawals returned to the baseline level and stayed at this level afterwards (93 mN: F_(4,28)_ = 3.72, *P*<0.05; 197 mN: F_(4,28)_ = 3.9, *P*<0.05). These results indicate that BM-MNC transplantation alleviated the persistence of mechanical hyperalgesia in diabetic rats.

**Figure 2:**
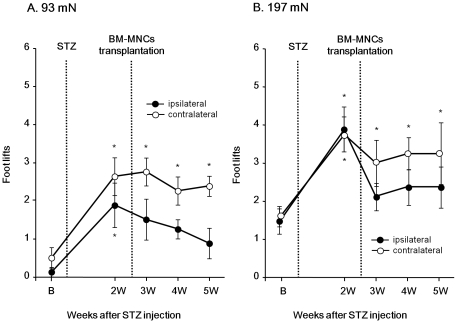
BM-MNC transplantation inhibited mechanical hyperalgesia in the ipsilateral diabetic hind paw. Number of foot withdrawals in response to the 93 (A) and 197 mN (B) von Frey hair (VFH) stimulation in both hind paws of diabetic rats are shown (n = 8). Data are presented as mean ± SEM. The form of presentation is the same as in Fig. 1. BM-MNC injection was carried out just after the measurements at 2 weeks (2W) after the STZ injection. Repeated measures two-way ANOVA identified a significant difference in the day-effect between both hind paws. *P<0.05 compared with before (B) STZ injection (one-way ANOVA with Dunnett's tests).

Figure 2 shows the effects of BM-MNC transplantation in the unilateral hindlimb muscles on mechanical hyperalgesia in the bilateral hind paws of the diabetic rats. As shown in Figure 1, the withdrawal frequencies of both bilateral hind paws to 93- and 197-mN VFH were significantly increased 2 weeks after STZ injection (93 mN: ipsilateral: F_(4,28)_ = 2.76, *P*<0.05; contralateral: F_(4,28)_ = 7.62, *P*<0.01; 197 mN: ipsilateral: F_(4,28)_ = 2.76, *P*<0.05; contralateral: F_(4,28)_ = 7.62, *P*<0.01). After BM-MNC transplantation, the STZ-induced increase in the withdrawal frequencies in the ipsilateral side was significantly inhibited. On the other hand, the withdrawal frequencies in the contralateral side remained at a high level for another 3 weeks. Repeated measures two-way ANOVA identified a significant difference in the day-effect between both hind paws (93 mN: group, F_(1,14)_ = 11.7, *P*<0.005; day, F_(4,56)_ = 6.96, *P*<0.001; interaction, F_(4,56)_ = 0.66, *P*>0.05; 197 mN: group, F_(1,14)_ = 1.95, *P*>0.05; day, F_(4,56)_ = 7.38, *P*<0.001; interaction, F_(4,56)_ = 0.68, *P*>0.05). These results indicate that BM-MNC transplantation in the unilateral hindlimb muscles inhibited mechanical hyperalgesia only in the ipsilateral hind paw of diabetic rats.

### Effects of BM-MNCs on cold allodynia

**Figure 3:**
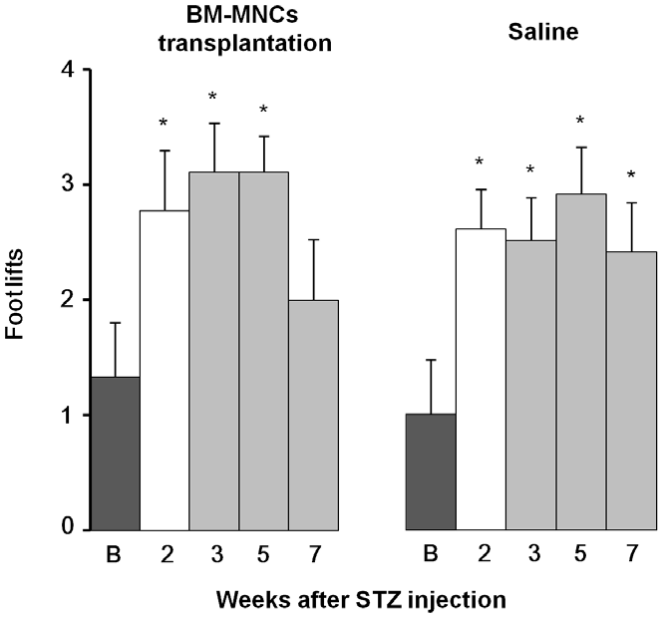
Inhibitory effects of BM-MNC transplantation on cold allodynia induced by STZ administration. Number of foot withdrawals in response to the acetone application in diabetic rats is shown. Data are presented as mean ± SEM. Vertical axis, number of foot lifts; horizontal axis, order of the test (weeks after STZ injection). Black column, before STZ injection; white column, 2 weeks after STZ injection; shadow column, 3, 5, and 7 weeks after STZ injection. In the treatment group (left panel), BM-MNC injection was carried out just after the measurements at 2 weeks after the STZ injection (n = 9). In the control group (right panel), saline injection was done (n = 10). *P<0.05 compared with before (B) STZ injection (one-way ANOVA with Dunnett's tests).

The effects of BM-MNC transplantation on the cold allodynia in the ipsilateral hind paw of diabetic rats are shown in Figure 3. The number of foot withdrawals in response to the acetone application in the diabetic control rats was significantly increased 2 weeks after STZ injection and remained high for another 5 weeks (Saline group, F_(4,36)_ = 5.83, *P*<0.001), implying that STZ-induced diabetes showed long-lasting cold allodynia. In the experimental group of rats, BM-MNC transplantation was done 2 weeks after the STZ injection. In this case, the increased withdrawal frequency in the diabetic rats was reduced to the baseline level 5 weeks after the transplantation (F_(4,32)_ = 3.55, *P*<0.05). These results indicate that BM-MNC transplantation also ameliorated the persistence of cold allodynia in diabetes, although the effect occurred later than that of mechanical hyperalgesia.

### Sciatic nerve conduction velocities

**Figure 4:**
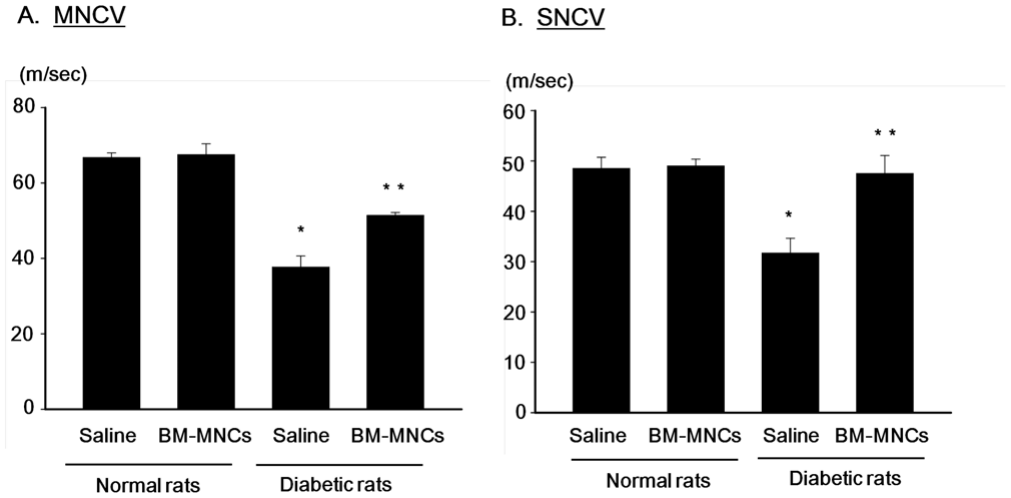
Effects of BM-MNC transplantation on sciatic motor nerve conduction velocity and sensory nerve conduction velocity. BM-MNCs were transplanted into unilateral hindlimb skeletal muscles 2 weeks after the STZ injection, and sciatic motor nerve conduction velocity (MNCV) and sensory nerve conduction velocity (SNCV) were measured 2 weeks later. Results are means ± SEM. *P<0.001 vs. saline-injected side of control rats. **P<0.01 vs. saline-injected side of diabetic rats.

Four weeks after the STZ and saline injections, changes in sciatic nerve conduction velocities in diabetic and normal rats were measured. In the saline-injected side of the diabetic rats, MNCV and SNCV were 36.4±2.6 m/s and 31.7±2.9 m/s, respectively, which were significantly reduced compared with those in the saline-injected side of normal rats (MNCV: 62.6±6.2 m/s, SNCV: 48.5±2.2 m/s, *P*<0.001 for both) (Figure 4). In diabetic rats, both MNCV and SNCV in the BM-MNC-injected side were significantly ameliorated (MNCV: 51.4±0.8 m/s, SNCV: 47.5±3.6 m/s). Transplantation of BM-MNCs in normal rats did not affect the sciatic nerve conduction velocities.

### Sciatic nerve blood flow (SNBF)

**Figure 5:**
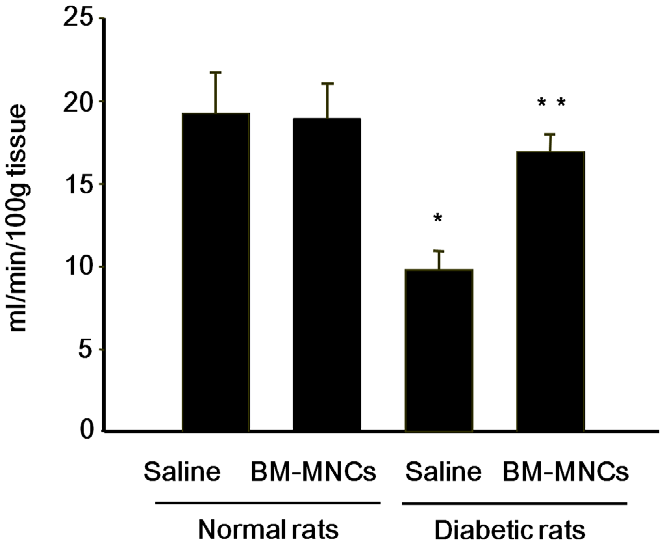
Effects of BM-MNC transplantation on sciatic nerve blood flow (SNBF). BM-MNCs were transplanted into unilateral hindlimb skeletal muscles 2 weeks after the STZ injection, and SNBF was measured 2 weeks later. Results are means ± SEM. *P<0.05 vs. saline-injected side of control rats. **P<0.01 vs. saline-injected side of diabetic rats.

Changes in SNBF in diabetic and control rats were also measured 4 weeks after the STZ and saline injections. As shown in Figure 5, SNBF was significantly decreased in the saline-injected side of diabetic rats (9.8±1.1 ml/min/100 g) compared with those in the saline-injected side of normal rats (19.2±2.5 ml/min/100 g, *P*<0.05), indicating that SNBF was decreased in the diabetic condition. In diabetic rats, SNBF in the BM-MNC-injected side (16.9±1.3 ml/min/100 g) was significantly increased compared with that in the saline-injected side (*P*<0.01). Transplantation of BM-MNCs in normal rats did not show significant changes of SNBF.

### Capillary density of skeletal muscles

**Figure 6:**
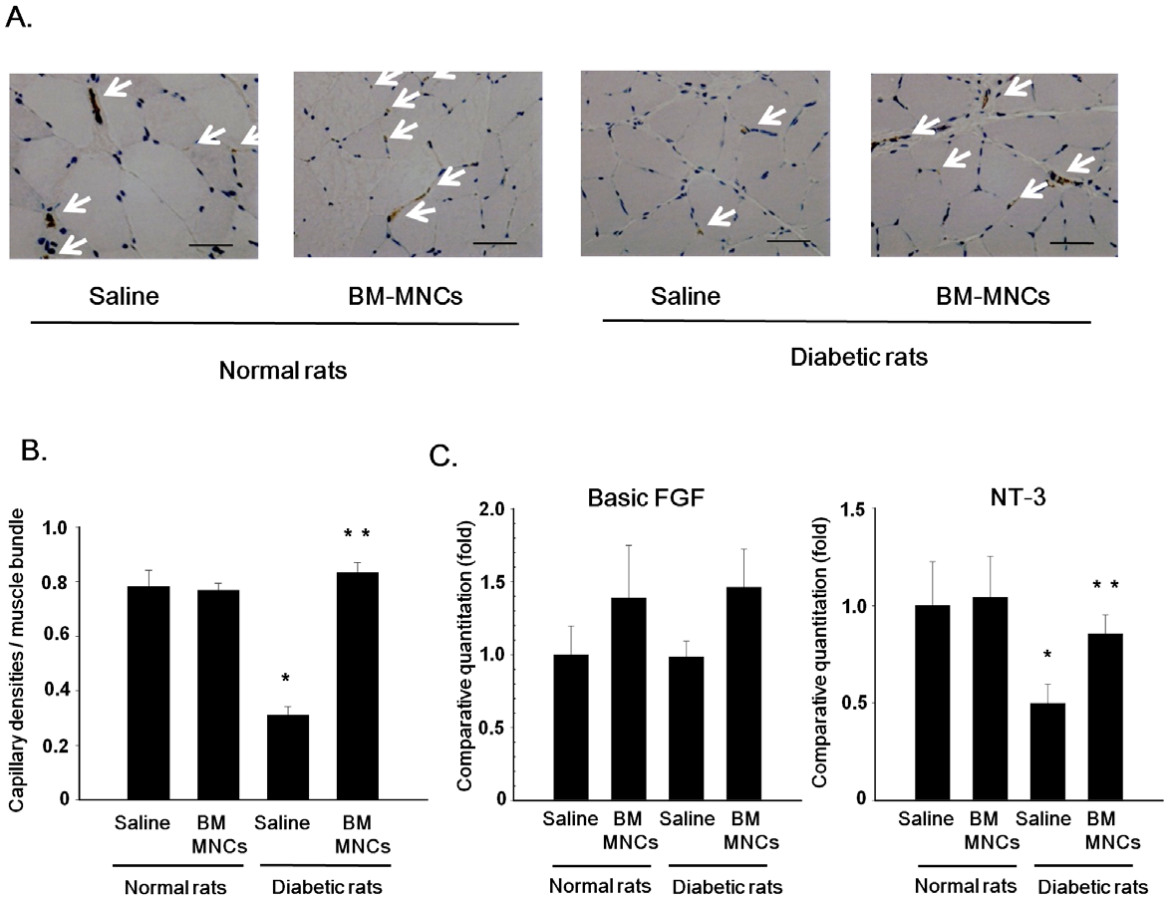
Capillary density and gene expressions in skeletal muscles. **A:** Representative photomicrographs of histological sections in the saline-injected and BM-MNC-injected sides of the skeletal muscles of normal and diabetic rats. Arrowheads indicate vascular endothelial cells detected by immunostaining for vWF. Bar = 50 µm. **B:** Quantitative analyses for capillary/muscle fiber ratio of the saline-injected and BM-MNC-injected sides of the skeletal muscles in normal and diabetic rats. Results are means ± SEM. *P<0.001 vs. saline-injected side of control rats. **P<0.001 vs. saline-injected side of diabetic rats. **C:** bFGF and NT-3 mRNA expressions in saline and BM-MNC-injected muscle. Results are means ± SEM. *P<0.05 vs. saline-injected side of control rats. **P<0.05 vs. saline-injected side of diabetic rats.

Vasculatures were visualized by vWF immunostaining, a specific marker for endothelial cells. As shown in Figure 6A, the capillary density was reduced in the saline-injected side of the diabetic rats. Quantitative analyses revealed that the capillary/muscle ratio in the saline-injected side of diabetic rats was significantly reduced compared with that in the saline-injected side of normal rats (Figure 6B, *P*<0.001). In diabetic rats, transplantation of BM-MNCs significantly increased the number of capillaries in skeletal muscles compared with those in the saline-injected side (*P*<0.001). Transplantation of BM-MNCs in normal rats did not affect the capillary/muscle ratio.

### Local gene expressions in skeletal muscles

The mRNA expression of NT-3 was significantly decreased in saline-injected side of the soleus muscles of diabetic rats (*P*<0.05) (Figure 6C). BM-MNC transplantation significantly increased the NT-3 mRNA expressions by 1.7 times compared with the saline-injected side of diabetic rats (*P*<0.05). BM-MNC transplantation tends to increase the bFGF mRNA expressions in the transplanted-side of diabetic rats, although the increase was not significant.

### Intraepidermal nerve fiver density (IENFD)

**Figure 7:**
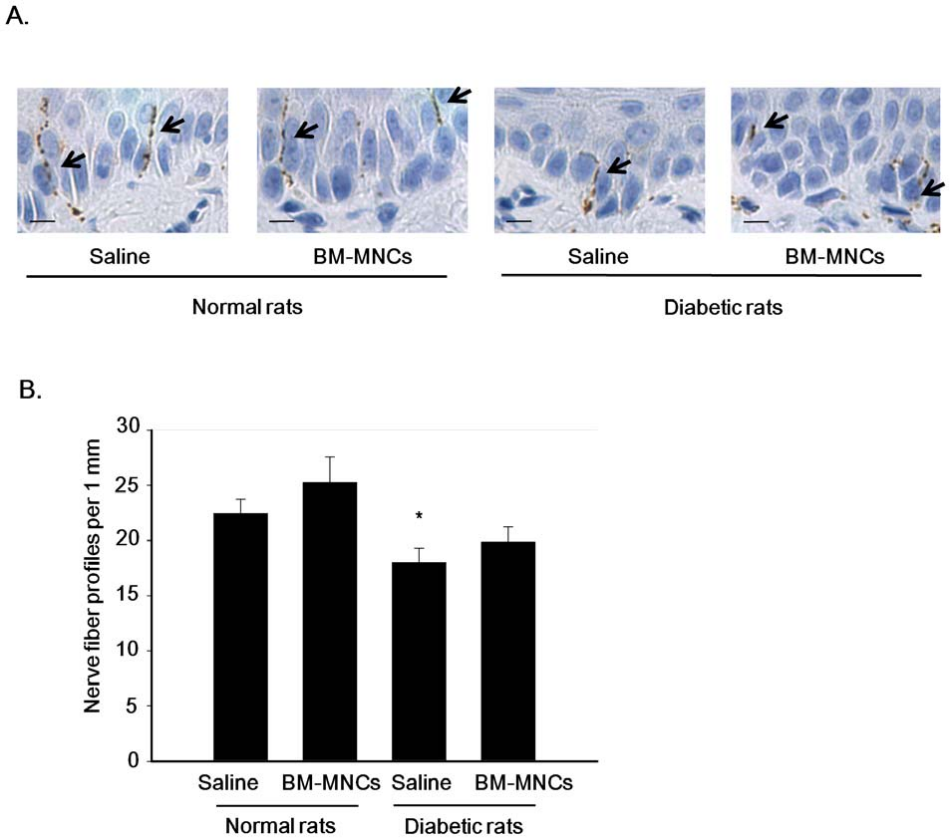
Intraepidermal nerve fiver density (IENFD). **A:** Representative photomicrographs of histological sections in the saline-injected and BM-MNC-injected sides of footpads of normal and diabetic rats. Arrowheads indicate intraepidermal nerve fiber detected by immunostaining for PGP 9.5. Bar = 10 µm. **B:** Quantitative analyses for IENFD in footpads of the saline-injected and BM-MNCs-injected sides in normal and diabetic rats. Results are means ± SEM. *P<0.05 vs. saline-injected side of control rats.

Nerve fibers were visualized by PGP9.5 immunostaining, a specific neuronal marker (Figure 7A). Quantitative analyses revealed that IENFD in the saline-injected side of the diabetic rats was significantly reduced compared with that in the saline-injected side of normal rats (saline-injected side of diabetic rats: 17.9±1.3/mm, saline-injected side of control rats: 22.4±1.3/mm, *P*<0.05) (Figure 7B). This implies that the 4-week duration of diabetes decreased the intraepidermal nerve fiber profiles (IENFD). BM-MNC transplantation tended to increase the IENFD in diabetic rats, but the change was not significant. BM-MNC transplantation had no significant effect in normal rats.

## Discussion

The present study first demonstrated that the transplantation of freshly isolated BM-MNCs into the hindlimb skeletal muscles alleviated the persistence of neuropathic pain (mechanical hyperalgesia and cold allodynia) in diabetic rats. The BM-MNC transplantation also improved the delay of sciatic nerve conduction velocities (MNCV and SNCV) in the diabetic rats. Our results, therefore, suggest that the transplantation of isolated BM-MNCs into the hindlimb skeletal muscles ameliorated the diabetic neuropathic pain as a result of the rescue of neural functions.

Painful diabetic neuropathy reduces the quality of life in diabetic patients. Most pharmacological managements of painful diabetic neuropathy are intended to provide relief from pain without any effects on the underlying causes and, in many cases, clinicians provide these pharmacological agents for relief from pain [28]. The symptoms are commonly described as prickling, knife-like, electric shock-like, burning, freezing together with hyperalgesia and allodynia [1]. Here, we demonstrated that BM-MNC transplantation ameliorated diabetic hyperalgesia and cold allodynia together with improvement of the nerve functions. Our results suggest that BM-MNC transplantation could become a new strategy for treating diabetic neuropathy.

Impairment of peripheral blood flow is one of the major factors in diabetic neuropathy. Our present study showed that the capillary/muscle ratio in the soleus muscle was significantly lower in the diabetic rats compared with normal rats, which is consistent with previous animal and human studies [19], [29]. The immunohistological study revealed that the BM-MNC transplantation increased the number of microvessels in the ipsilateral soleus muscle. In the sciatic nerve, STZ-induced diabetes induced a reduction of the nerve blood flow and this deficit was recovered by BM-MNC transplantation. Kim et al. indicated that transplanted BM-MNCs preferentially engrafted in the sciatic nerve and improved nerve blood flow [22]. These results, therefore, suggest that improvement of the blood flow in the tissues including nerve vessels is one of the crucial effects of BM-MNC transplantation. Clinical trials of the transplantation of MNCs have mainly been conducted for the rescue of ischemic tissues, such as from myocardial infarction and atherosclerosis obliterans [15], [16], [30]. It has been demonstrated that one of the mechanisms mediating the effects of MNC transplantation is the collateral formation in the ischemic tissue by the abundant secretion of cytokines, such as VEGF and bFGF, that play a crucial role in neovascularization [31]. Our present study showed that transplantation of BM-MNCs into the unilateral hindlimb skeletal muscles inhibited mechanical hyperalgesia in the ipsilateral side but not in the contralateral side. Two clinical studies supported these observations linking painful diabetic neuropathy with alterations in blood flow, and demonstrated significant benefits of pain relief from the use of vasodilators, isosorbide dinitrate spray, and glyceryl trinitrate patches [32], [33].

bFGF is also a neuroprotective cytokine [34], [35], and we previously revealed that intramuscular injection of bFGF with cross-linked gelatin hydrogel improved the sciatic nerve conduction velocity, hypoalgesia, and sciatic nerve blood flow in STZ-induced diabetic rats [36]. In this study, we demonstrated that BM-MNC transplantation increased the gene expressions of bFGF and NT-3 in the BM-MNC-transplanted site of skeletal muscles in diabetic rats. NT-3 works as neurotrophic factor [37]. A previous study reported the reduction of chronic constriction injury-induced neuropathic pain by the attenuation of neuronal expression of the sodium channel [38]. A previous report revealed that transplantation of BM-MNCs into diabetic hindlimb skeletal muscles increased several gene expressions such as VEGF, bFGF and IGF-1 in sciatic nerve. IGF-1 is also reported to ameliorate neuropathic pain and diabetic hyperalgesia [39]. A recent study revealed that BM-MNC transplantation into ischemic heart normalized more than 2099 genes altered by myocardial infarction and changed more than 200 gene expressions which were independent of ischemia using microarray analysis, indicating that BM-MNC transplantation affects many gene expressions [40]. These results suggest that the effects of BM-MNC transplantation on diabetic hyperalgesia and allodynia are achieved not by a single mechanism, but by multifocal mechanisms depending on changes in growth factors, neurotrophic factors and other multiple gene expressions.

It is interesting that there is a difference in the onset of efficacy on mechanical hyperalgesia and cold allodynia by BM-MNC transplantation. BM-MNCs ameliorated mechanical hyperalgesia within 1 week after transplantation, whereas, it took 5 weeks to improve cold allodynia after transplantation. This discrepancy in the efficacy is often observed in the treatment of painful diabetic neuropathy [41], [42]. Several human and animal studies demonstrated that small unmyelinated (C) fibers and thinly myelinated (A-δ) fibers as well as myelinated large (A-β) fibers, are damaged in the diabetic neuropathic condition [43], [44]. Our skin biopsy study revealed that IENFD in the footpad, indicating the amount of C fiber sensory units [45], was decreased 4 weeks after the induction of diabetes, whereas both mechanical hyperalgesia and cold allodynia continued. These results indicate that these painful behaviors were not due to decreased IENFD (i.e., C-fibers), but to functional changes in the nerve fibers. We previously found in *in vitro* experiments using skin-saphenous nerve preparations taken from rats in the early stage of STZ-induced diabetes that C-fiber nociceptors in the skin showed increased spontaneous activity and a lowered response threshold to mechanical stimulation under perfusion with high-glucose solution [46]. In normal rats, on the other hand, these augmenting effects of high-glucose were not observed. These results suggest that C-fiber nociceptors that had been spared serious damage by the diabetes play an important role in the aggravation of pain in diabetic neuropathy. Fuchs et al. revealed that sensory and neurosecretory nociceptor functions are sensitized in diabetic rats and hyperglycemia with hypoxia increased the sensitivity of diabetic C-fibers more than hyperglycemia alone [47] suggesting that not only the correction of diabetes but also the recovery from hypoxia is a valuable target for the treatment of painful diabetic neuropathy. On the other hand, it has been demonstrated that hypersensitivity to mechanical stimulation persisted even when C-fiber responses were abolished with resiniferatoxin [48]. This indicates that hypersensitivity to mechanical stimulation in diabetic rats is mediated by larger myelinated (A-β and A-δ) fibers rather than by C-fibers. The fact that vasodilator treatment or drugs that increase the nerve blood flow ameliorated pain-related behaviors and improved the slowed nerve conduction velocity [49], [50] may indicate that larger myelinated (A-β and A-δ) fibers might also play an important role in diabetic pain. Taken together, the multifocal efficacy of BM-MNC transplantation may be a favorable treatment for painful diabetic neuropathy.

An important benefit of BM-MNC transplantation is that BM-MNCs can be isolated in a closed cavity, in contrast to endothelial progenitor cells and mesenchymal stem cells that require *ex vivo* culture. Clinical trials for ischemic diseases using BM-MNCs revealed their safety and efficiency [15], [16]. However, future clinical study is required to identify the effects of BM-MNC transplantation on painful diabetic neuropathy.

We did not evaluate the effects of BM-MNC transplantation on neuropeptides in our study. We investigated diabetic hyperalgesia in the early stage diabetic neuropathy. We evaluated each measurement around 4 weeks after STZ injection in SD rats. A previous report revealed that the basal levels of neuropeptides such as calcitonin gene-related peptide (CGRP), substance P (SP) and prostaglandin E_2_ (PGE_2_) were reduced or not changed between the control and diabetic hyperalgesia group, but greater release of these pain-related peptides was observed by chemical stimulation with bradykinin in a diabetic skin-nerve preparation than in control [51]. In addition, we showed mechanical hyperalgesia and cold allodynia without an increase of the nerve fiber density in the skin. These results suggest that the neural hypersensitivity is the main phenomenon in the early stage of diabetic neuropathy, though further study is required to address this question.

In summary, we demonstrated that the transplantation of BM-MNCs ameliorated the diabetic neuropathic pain, caused functional recovery of the peripheral nerves and increased nerve blood flow. To our knowledge, this is the first report that BM-MNC transplantation ameliorated mechanical hyperalgesia and cold allodynia in diabetic neuropathy. The autologous transplantation of BM-MNCs could be a novel strategy for the treatment of painful diabetic neuropathy.

## Supporting Information

Table S1 **Body weights and blood glucose concentrations of SD rats.**
(DOC)
