# Supplementary material for: Transplantation of Bone Marrow-Derived Mononuclear Cells Improves Mechanical Hyperalgesia, Cold Allodynia and Nerve Function in Diabetic Neuropathy
Source: PLoS One. 2011 Nov 18;6(11):e27458. doi: 10.1371/journal.pone.0027458 (PMC3220696; doi:10.1371/journal.pone.0027458)
Supplement: Table S1 — Body weights and blood glucose concentrations of SD rats. (DOC) [file pone.0027458.s001.doc]

**Table S1**.

Body weights and blood glucose concentrations of SD rats.

|  | Body weights  (g) | Blood glucose (mM) |
| --- | --- | --- |
| Normal rats with BM-MNC transplantation | 341 ± 3.6 | 5.9 ± 0.2 |
| Diabetic rats with BM-MNC transplantation | 255 ± 8.4* | 22.8 ± 1.3* |
| Diabetic rats without transplantation | 251 ± 14.9* | 23.2 ± 2.3* |

Data are expressed as means ± SEM (^*^*P*<0.001 versus normal rats).
